# Causal Associations of Circulating Micronutrients With the Risk of Infertility: A Mendelian Randomization Study

**DOI:** 10.1002/fsn3.71084

**Published:** 2025-10-28

**Authors:** Jiaxin Zhang, Bo Hu, Tao Wang, Pusheng Yang, Xiaotong Peng, Yaxin Miao, Wenwen Liu, Xin Lin, Jing Sun

**Affiliations:** ^1^ Shanghai Key Laboratory of Maternal Fetal Medicine, Shanghai Institute of Maternal‐Fetal Medicine and Gynecologic Oncology, Shanghai First Maternity and Infant Hospital, School of Medicine Tongji University Shanghai People's Republic of China; ^2^ Nanomedicine and Intestinal Microecology Research Center, Shanghai Tenth People's Hospital, School of Medicine Tongji University Shanghai People's Republic of China; ^3^ Department of Chemoradiotherapy the Affiliated People's Hospital of Ningbo University Ningbo China

**Keywords:** β‐carotene, infertility, iron, mendelian randomization, micronutrients, phosphatase, selenium

## Abstract

Infertility impacts 48 million couples globally, and accumulating evidence suggests that micronutrients potentially influence reproductive health. This Mendelian randomization study investigates causal relationships between 15 micronutrients and infertility in both males and females, aiming to complement existing nutritional epidemiology insights. Genetic association estimates for micronutrient biomarkers (including selenium, iron, β‐carotene, calcium, magnesium, phosphorus, folate, vitamins B_6_, B_12_, C, D, zinc, copper, iodine, and manganese) and infertility phenotypes were derived from European‐ancestry genome‐wide association study (GWAS) cohorts. For causal inference, inverse‐variance weighted MR served as the primary analytical method, supplemented by MR‐Egger and weighted median approaches. In female, genetically predicted higher levels of selenium (OR = 0.94; 95% CI = 0.90–0.99; *p* = 0.019), iron (OR = 0.89; 95% CI = 0.80–0.98; *p* = 0.023), and β‐carotene (OR = 0.87; 95% CI = 0.80–0.96; *p* = 0.005) demonstrated inverse associations with risk, suggesting potential protective effects. In males, higher phosphorus exhibited a strong positive correlation with infertility (OR = 4.05; 95% CI = 1.37–11.96; *p* = 0.011). No significant associations were observed for the remaining micronutrients. This Mendelian randomization study comprehensively evaluates the causal effects of 15 micronutrients on infertility in both sexes. The findings highlight potential protective roles of selenium, iron, and β‐carotene in female infertility and identify phosphorus as a risk factor for male infertility. These results support the development of sex‐specific nutritional strategies for fertility improvement.

## Graphical abstract

This Mendelian randomization study revealed that higher genetically predicted levels of selenium, iron, and β‐carotene are associated with a reduced risk of female infertility, while elevated phosphorus levels are positively linked to male infertility. These findings suggest sex‐specific micronutrient influences on reproductive health and support the development of targeted nutritional strategies.

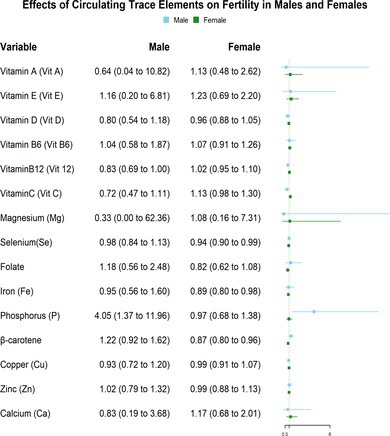

## 1 Introduction

Infertility, clinically defined as the failure to achieve a clinical pregnancy after 12 months or more of regular unprotected intercourse, affects approximately 48 million couples worldwide (Suks et al. 2021). According to World Health Organization (WHO) reports, the prevalence of infertility ranges between 8% and 12% across diverse populations (Mélodie and Christine 2018; Robert 2006). Among these cases, female factors contribute to 35% of cases, male factors to 50%, and 15% are classified as “unexplained infertility” (Skoracka et al. 2020). This complexity reflects the involvement of complex interactions among genetic predispositions, environmental exposures, lifestyle influences, and micronutrient homeostasis (Sulagna et al. 2021).

Modifiable risks—such as dietary deficiencies, oxidative stress, and environmental pollutant exposure—are increasingly recognized for their roles in disrupting gamete quality, hormonal regulation, and embryonic development (Benatta et al. 2020; Lakoma et al. 2023). Although required in small amounts, micronutrients serve as essential cofactors in enzymatic reactions that regulate redox balance, metabolism, and endocrine function (Hauser et al. 2016). There has been sufficient evidence on the significance of nutrition in fertility, particularly micronutrients such as multivitamins and minerals (Brooke et al. 2017). Deficiencies in essential micronutrients may damage fertility and compromise pregnancy outcomes, whereas excessive intake of certain micronutrients may exert toxic effects (Gonzalez‐Martin et al. 2024).

Clinical evidence suggests the role of nutritional interventions: antioxidant supplementation (e.g., selenium, vitamin E) can modestly improve clinical pregnancy rates in subfertile women from 19% to 25%–30% (Showell et al. 2020), and increased intake of β‐carotene or vitamin C may shorten time‐to‐pregnancy in specific subgroups (Ruder et al. 2014). However, observational studies and randomized controlled trials (RCTs) have yielded inconsistent results. For example, while an observational study suggested that vitamin D has been associated with PCOS and endometriosis, RCTs have not shown consistent effects on fecundability (Polyzos et al. 2014; Simpson and Pal 2023).

Such discrepancies likely arise from confounding biases inherent in observational study designs, variability in biomarker measurements, and ethical and logistical challenges of conducting long‐term nutritional randomized controlled trials. To address these limitations, alternative approaches are needed. Mendelian randomization (MR), an epidemiological method utilizing genetic variants as instrumental variables (IVs) for modifiable exposures, circumvents these challenges by leveraging the random allocation of alleles during meiosis, thereby approximating the conditions of a randomized trial (Zaixiang et al. 2024). By minimizing confounding and reverse causation, MR provides robust estimates of causal relationships.

Applying this approach, we systematically investigated the causal effects of 15 circulating micronutrients—including vitamins A, B6, B12, C, D, E, β‐carotene, and minerals such as magnesium, selenium, folate, iron, phosphorus, copper, zinc, and calcium—on the infertility risk of both sexes. Genetic instruments for these micronutrients were obtained from large‐scale genome‐wide association studies (GWAS), ensuring robust statistical power. The study aims to determine whether genetically predicted concentrations of these micronutrients influence infertility risk in both men and women, thereby contributing to the development of sex‐specific evidence‐based nutritional strategies for reproductive health (Ferrucci et al. 2009; Grarup et al. 2013; Kestenbaum et al. 2010; Major et al. 2011; Meyer et al. 2010; Mondul et al. 2011; O'Seaghdha et al. 2013; Tanaka et al. 2009).

## 2 Methods

### 2.1 Study Design

**FIGURE 1:**
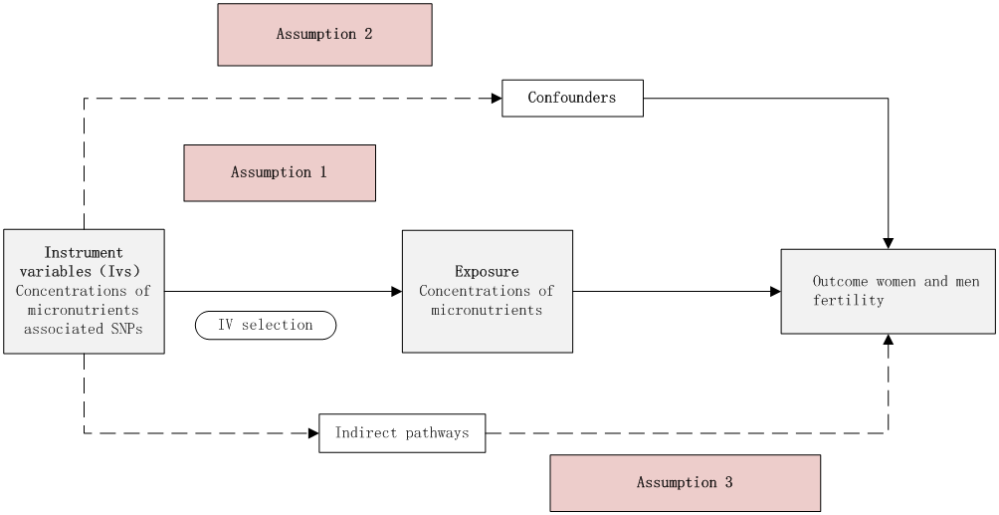
The conceptual Mendelian randomization framework.

The schematic overview of this study was presented in Figure 1. When the three key assumptions were met, the causal relationship between micro‐nutrients and infertility, Mendelian randomization analysis was used to examine the potential causal association between them: (1) The genetic variants are robustly associated with the exposure (micronutrient concentration). (2) The genetic variants are independent of potential confounding variables. (3) The variants influence only through the exposure to genetic variants to influence the outcome without involvement of another pathway.

### 2.2 Genetically Predicted Circulating Micronutrient Levels

We systematically selected 15 micronutrients prioritized based on: (1) biological relevance to human reproductive physiology, corroborated by prior mechanistic studies; (2) availability of genome‐wide association study (GWAS) summary statistics for European‐ancestry populations. These included vitamins A (retinol), B_6_, B_12_, C, D, E, β‐carotene, magnesium (Mg), selenium (Se), ionized calcium (Ca), iron (Fe), copper (Cu), zinc (Zn), phosphorus, and folate. Full GWAS metadata (sample sizes, consortium sources, variant counts) is detailed in Table 1.

**TABLE 1:** Source of exposure genome‐wide association study summary data.

Phenotype	Participants	Ancestry	Varienced explained	PMID	Number of SNPs available
Vit A	8902	Caucasians	2.3	21878437	2
Vit E	5006	Europeans	1.7	21729881	3
Vit D	122,123	Europeans	2.13	29343764	6
Vit B6	1864	Europeans	1	19303062	2
Vit B12	37,465	Europeans	6.3	23754956	10/7
Vit C	52,018	Europeans	1.79	33203707	10/9
Mg	15,366	Europeans	1.6	20700443	5
Se	9639	Europeans	6.90	25343990	11
Folate	37,465	Europeans	1	23754956	2
Fe	48,972	Europeans	3.4	25352340	3/2
Phosphorus	16,264	Europeans	1.5	20558539	4
β‐carotene	3918	Europeans	4.5	19185284	4
Cu	2603	Australians	5	23720494	2
Zn	2603	Australians	8	23720494	2
Ca	61,054	Europeans	0.9	24068962	7

Abbreviations: Ca, Calcium; Cu, Copper; Fe, iron; Mg, Magnesium; Se, Selenium; vit A, vitamin A; Vit B6, vitamin B6; Vit B12, vitamin B12; Vit C, vitamin C; Vit D, vitamin D; vit E, vitamin E; Zn, zinc.

### 2.3 Genetically Predicted Infertility Risk

Sex‐stratified infertility GWAS data were curated from the FinnGen Consortium Release 10 (https://r10.finngen.fi/), encompassing 6481 female cases (failure to conceive ≥ 12 months) versus 68,969 female controls, and 680 male cases versus 72,799 male controls (Table 2). All participants were of European ancestry, with phenotypes defined by ICD‐10 codes.

**TABLE 2:** Source of outcome genome‐wide association study summary data.

Phenotype	Participants	Ancestry	Year	ID
Female infertility	75,450	European	2021	finn‐b‐N14_FEMALEINFERT
Male infertility	73,479	European	2021	finn‐b‐N14_MALEINFERT

### 2.4 Selection of Instrumental Variables

Published GWAS for 15 nutrients were finally retrieved. In order to meet the first assumption, the *p* was set < 10E‐8. As for the second assumption, distance was set at 10,000 kb, and linkage disequilibrium was set at *r* ^2^ < 0.1, while for selenium, an *r* ^2^ < 0.3 was in line with previous studies. The strand direction consistency of SNPs was checked, and the palindromic SNP will be identified and subsequently removed. Additionally, a proxy SNP with high linkage disequilibrium (*R* ^2^ > 0.8) (available at SNiPA—a single nucleotide polymorphisms annotator and browser) will replace the missing SNPs, covering the shortage of the micronutrients, as the number was small (Table S1). All data used in this study were openly accessible, and appropriate consent and ethical approvals were obtained from relevant participants.

### 2.5 MR Analysis

There were five different MR methods used in the study to evaluate the relationship between micronutrient concentrations and infertility. Inverse‐variance weighted (IVW) is the primary analysis method based on the multiple random effects; the Wald ratio estimate was calculated by dividing the effect size for each micronutrient concentration SNPs. Weighted Median (WM), MR‐Egger, Weighted mode, and Simple mode methods were the alternative MR analyses; the scatter plots of each MR approach were used in MR analysis. The F statistic was calculated by (beta^2^/Se^2^). *F* value < 10 suggests the likelihood of weak instrumental variable bias.

### 2.6 Sensitivity Analyses

Additionally, leave‐one‐out tests were conducted to evaluate the validity and robustness of the results, and the funnel plots identified the potential horizontal pleiotropy. The horizontal pleiotropy tests between circulating micronutrients and infertility in men and women were performed by MR‐PRESSO methods. If there were any outliers detected, we removed them and corrected the outcomes. We performed Cochran's *Q* test to figure out the heterogeneity; a random‐effects model of the IVW method was employed if heterogeneity was identified; otherwise, we chose the fixed‐effects model. MR analysis estimates the association of micronutrients with the risk of female and male infertility.

### 2.7 Statistical Tools

All MR analyses were conducted using the Two‐Sample MR package (version 0.5.6) in R (version 4.0.3). If the threshold *p* < 0.05, it was considered that the statistically significant results were significant.

## 3 Result

**FIGURE 2:**
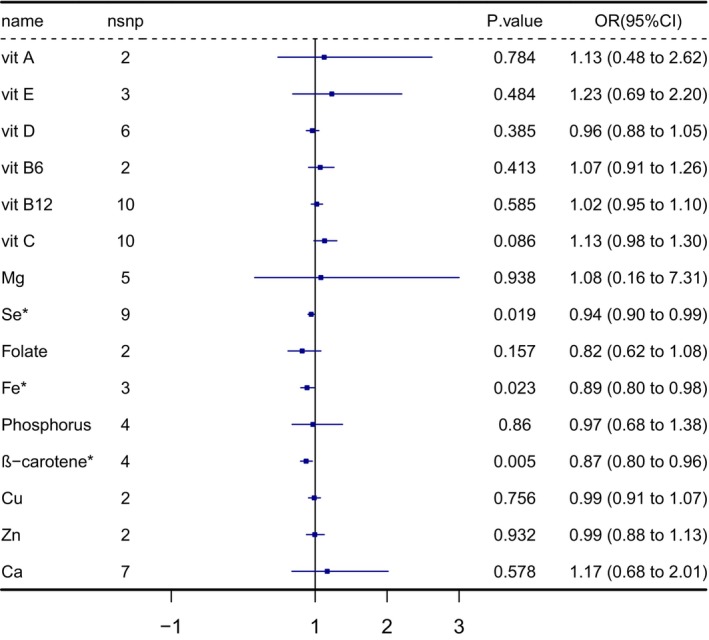
The IVW results of MR analysis studying the effect of 15 micronutrient concentrations on female infertility outcomes; * The IVW results for selenium, iron, and β‐carotene concentration effects on female infertility indicate statistical significance. Ca, calcium; Cu, copper; IVW, inverse variance weighted; Fe, iron; Mg, magnesium; MR, Mendelian randomization; OR, odds ratio; Se, selenium; vit A, vitamin A; vit B6, vitamin B6; vit B12, vitamin B12; vit C, vitamin C; vit D, vitamin D; vit E, vitamin E; Zn, zinc. For the significance and sensitivity analysis, Cochran's *Q* test and the MR‐Egger test suggested there was no heterogeneity and pleiotropy in the significant result (Table 3).

**FIGURE 3:**
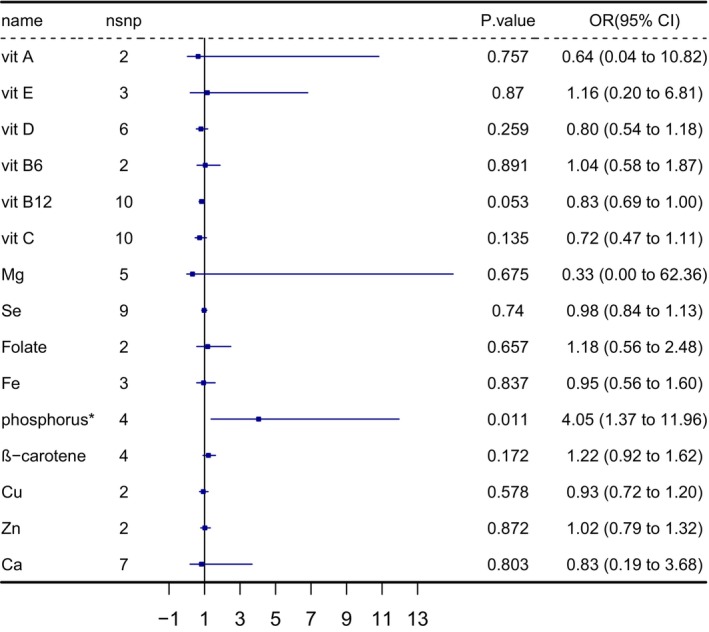
The IVW results of MR analysis studying the effect of 15 micronutrient concentrations on female infertility outcomes; * The IVW results concentration of phosphorus on male infertility indicate statistical significance. Ca, Calcium; Cu, Copper; Fe, iron; IVW, inverse variance weighted; Mg Magnesium; MR, Mendelian randomization; OR, odds ratio; vit A, vitamin A; vit E, vitamin E; vit D, vitamin D; vit B6, vitamin B6; vit B12, vitamin B12; vit C, vitamin C; Se, Selenium; Zn, zinc.

After a rigorous selection of genetic instruments, 75 SNPs were used to genetically predict 15 micronutrients associated with infertility in men and women. As the study results represented, serum selenium concentrations (odds ratio [OR] = 0.94; 95% confidence interval [CI] = 0.90–0.99, *p* = 0.019), serum iron (OR = 0.89; 95% CI = 0.80–0.98, *p* = 0.023), and serum β‐carotene (OR = 0.87; 95% CI = 0.80–0.96, *p* = 0.005) were negatively associated with the risk for female infertility, which was shown in Figure 2. As for male infertility, only serum phosphorus was positively related (OR = 4.05; 95% CI = 1.37–11.96, *p* = 0.011) to infertility (Figure 3). The weighted median analysis and weighted mode analysis of the above factors were also significant (*p* < 0.05), as Tables S2 and S3 represented.

For the significance and sensitivity analysis, Cochran's *Q* test and the MR‐Egger test suggested there was no heterogeneity and pleiotropy in the significant result (Table 3).

**TABLE 3:** Variation scale and heterogeneity analysis.

Exposure	Outcome	SNP	Heterogeneity tests		Directional horizontal pleiotropy (MR‐Egger)
			MR‐Egger	IVW	
Se	Female infertility	9	0.536	0.603	0.564
Fe	Female infertility	3	0.784	0.503	0.458
ß − carotene	Female infertility	4	0.665	0.827	0.806
Phosphorus	Male infertility	4	0.742	0.897	0.904

Abbreviations: Fe, iron; Se, Selenium.

The leave—one—out sensitivity analysis (Figure S1) showed relatively robust results, meaning that different SNP loci did not significantly interfere with the analysis results.

The funnel plots suggest that SNP loci have a certain impact on the effect estimates, and there is a certain degree of heterogeneity; the two Mendelian randomization analysis methods can provide relatively consistent judgments on the association trends in most cases, as shown in Figure S2. The scatter plots (Figure S3) show that multiple MR methods can, in most cases, provide consistent judgments on the association trends. Although some SNP loci can affect the effect estimates, they do not fundamentally change the overall association trends, and the results exhibit a certain degree of reliability and robustness. The forest plot results (Figure S4) indicate that the overall effects of these elements on infertility are not significant, so more studies are needed to confirm the results.

Finally, we conclude that the main results were a positive association between serum selenium, as well as serum concentrations of iron and β‐carotene, which were associated with female infertility in the same direction as selenium, and the serum phosphorus levels were negatively associated with male infertility.

## 4 Discussion

Our study employed Mendelian randomization (MR) analysis to reveal causal relationships between 15 micronutrients and infertility risks in both sexes. Genetically predicted higher concentrations of selenium, iron, and β‐carotene were significantly associated with reduced risks of female infertility. Conversely, elevated serum phosphorus exhibited a male‐specific adverse effect, while no significant associations were detected for other micronutrients in males.

Our findings elucidate the complex interplay between trace elements and infertility, providing valuable insights into their etiology and pathogenic mechanisms. These results underscore the necessity of sex‐specific precision strategies in nutritional epidemiology and reproductive health interventions.

### 4.1 Selenium: Reproductive Mechanisms and Clinical Insights

Selenium, an essential trace element, is critical for human health and reproductive functions within physiological intake ranges (Mojadadi et al. 2021). Mendelian randomization (MR) studies have identified selenium as a protective factor for female fertility, while showing no discernible causal effect in males.

In females, selenium safeguards reproductive health through three synergistic pathways: ovarian protection, modulation of pregnancy outcomes, and regulation of thyroid‐related infertility. During late follicular development, expression of selenium uptake receptor LRP8 and antioxidant selenoprotein GPX1 positively correlated with steroidogenic enzymes CYP11A1, CYP19A1, and their electron transport chain components (FDXR, FDX1, POR), suggesting that granulosa cells depend on selenium and GPX1 to counteract oxidative stress during steroidogenesis, thereby preserving oocyte quality (Hummitzsch et al. 2024). Sodium selenite supplementation (5 ng/mL) has been shown to promote oocyte growth and enhance the proliferation of theca and granulosa cells, partly through inhibition of nitric oxide (NO)‐induced DNA damage (Basini and Tamanini 2000). In porcine parthenogenetic embryos, sodium selenite supplementation increased blastocyst formation rates, total cell number, and the inner cell mass ratio (Uhm et al. 2007).

In obstetrics, selenium deficiency reduces selenoprotein‐mediated redox regulation, impairing placental function and fetal growth, which may lead to miscarriage or complicated preterm birth (Duntas 2020; Hofstee et al. 2019). Clinically, selenium supplementation (200 μg/day sodium selenite) in pregnant women at risk of intrauterine growth restriction has been associated with reduced complications, including preterm birth and preeclampsia (Mesdaghinia et al. 2017).

Regarding thyroid‐related infertility, selenium supplementation (200 μg/day sodium selenite) can reduce TPOAb levels by 40%, improve thyroid function, and thereby enhance fertility outcomes (Gärtner et al. 2002). Selenoproteins such as DIO3, a key deiodinase, are critical for regulating thyroid hormone availability in the uterus and placenta, protecting the fetus from excessive maternal thyroid hormone exposure, and supporting normal pregnancy (Huang et al. 2003).

In males, selenoprotein GPX4 theoretically protects germ cells from oxidative DNA damage and stabilizes the sperm midpiece and acrosome, thereby supporting motility and fertilization (Safarinejad and Safarinejad 2009; Ursini et al. 1999). Although selenoprotein genes are located on autosomes, selenium metabolism exhibits sex‐specific patterns due to differences in tissue‐specific expression, hormonal regulation, and metabolic kinetics. The highly expressed SelP and ApoER2 are present in the testes of males, while they are marginally present or absent in the female ovary and uterus (Barchielli et al. 2022). Testosterone promotes selenoprotein synthesis in males, while estrogen modulates selenium handling in females, with lower reproductive tissue selenium retention (Schomburg 2016; Seale et al. 2018).

Despite the mechanistic importance of GPX4, a trial supplementing selenium yeast to men for 48 weeks showed no significant improvements in sperm parameters (Hawkes et al. 2009). In contrast, selenium supplementation in women with occult primary ovarian insufficiency (OPOI) improved AMH, antral follicle count, and ovarian volume without adverse effects, supporting targeted selenium optimization for female ovarian health (Safiyeh et al. 2021).

Our MR findings—showing no causal link between selenium and male infertility—may seem inconsistent with mechanistic evidence. However, this may reflect a threshold effect, where typical selenium intake already ensures adequate GPX4 activity, and tight testicular selenium regulation buffers against variation. In conclusion, while selenium is essential for male reproductive biology, additional selenium may offer limited benefits in selenium‐replete populations, whereas women with lower baseline selenium utilization may benefit more from targeted selenium optimization.

### 4.2 Iron: From Systemic Stores to Ovarian Health

Previous studies have highlighted a potential association between low serum ferritin (iron deficiency) and reproductive dysfunction in women: A cohort study reported that women with recurrent pregnancy loss (RPL) had significantly lower serum ferritin levels compared to controls (39.9 μg/L vs. 62.2 μg/L) and a higher prevalence of low iron stores (serum ferritin < 30 μg/L; 35.7% vs. 13.7%) (Georgsen et al. 2021). One plausible mechanism is that adequate iron status supports ovulation and follicular development. A large cohort study demonstrated that women who consumed iron supplements had a significantly lower risk of ovulatory infertility compared to non‐supplement users (relative risk 0.60, 95% CI 0.39–0.92) (Rushton et al. 1991). Similarly, in a prospective cohort study involving 18,555 premenopausal women, iron supplementation and higher non‐heme iron intake (primarily from multivitamins and iron supplements) were associated with a significantly reduced risk of ovulatory infertility (adjusted relative risk = 0.60, *p* = 0.005) (Chavarro et al. 2006). Iron deficiency arrested mouse estrous cycles at diestrus, blocked secondary follicular development and ovulation due to reduced ovarian ATP levels, downregulation of follicular development markers (FSHR, CYP19A1, CCND2), and decreased estradiol—17β (E2) compared to controls. Notably, iron repletion for 3 weeks reversed these impairments, restoring normal estrous cyclicity and fertility, highlighting that iron deficiency impairs ovarian function via disrupted energy metabolism and gene expression, and these effects are fully reversible via iron repletion (Tonai et al. 2020). Moreover, a study demonstrated heterogeneous expression of transferrin (Tf) and its receptor (TfR1) in human ovarian granulosa cells, with significantly higher levels in more mature follicles. Follicular fluid Tf concentrations strongly correlated with serum levels (*r* = 0.82, *p* < 0.001), suggesting Tf may contribute to follicular development through both systemic iron supply and granulosa‐derived biosynthesis. This interplay likely reflected iron‐dependent metabolic reprogramming critical for follicle maturation (Briggs et al. 1999).

### 4.3 β—Carotene: Divergent Roles in Female and Male Fertility

The results of this analysis showed that β‐carotene is positively correlated with female fertility, but demonstrated no association with male fertility. This finding aligns with certain existing studies while diverging from others, warranting further discussion.

From the perspective of female fertility, our results are consistent with numerous prior investigations. Several studies have indicated that β—carotene plays a beneficial role in the female reproductive process: Among women with one or two pregnancy losses, higher β—carotene levels have been linked to a shorter time to subsequent pregnancy. More broadly, greater β—carotene intake is linked to a shorter time to pregnancy, particularly in the population with a BMI ≥ 25 and age < 35 years old (Kim et al. 2018; Ruder et al. 2014). As an antioxidant, β‐carotene enhances female fertility through multiple mechanisms. Under in vitro oxidative stress conditions, β‐carotene has been shown to promote the development and maturation of oocytes. Proposed pathways include reducing reactive oxygen species (ROS) production and apoptosis, restoring the actin expression, facilitating the formation of the cortical granule‐free domain (CGFD), ensuring uniform mitochondria distribution, and promoting nuclear maturation. Animal experiments have further demonstrated that β‐carotene improves mitochondrial function and reduces oxidative damage to eggs (Yu et al. 2019). This series of studies explains the possible reasons for the positive correlation between β‐carotene and female fertility from different aspects and also provides strong theoretical support for our Mendelian randomization analysis results.

However, when focusing on male fertility, our analysis indicates that β‐carotene shows no significant association, which contradicts findings from some previous studies. Previous cross‐sectional studies have reported that, among men from infertile couples as well as young male university students, dietary β‐carotene intake is positively associated with total motile sperm count (TMSC) (De Cosmi et al. 2021; Mínguez‐Alarcón et al. 2012). Moreover, healthy dietary patterns characterized by a high intake of antioxidant micronutrients—including β‐carotene—and low levels of saturated and trans fatty acids are inversely correlated with poor semen‐quality parameters.

In conclusion, the positive correlation between β‐carotene and female fertility in our Mendelian randomization analysis results has been supported by various existing studies, while the result that β‐carotene has no association with male fertility, although inconsistent with some studies, can be explained by differences in research samples, methods, and indicators (Salas‐Huetos et al. 2017).

The discrepancies between our findings and those of previous observational studies can be interpreted from three perspectives. First, methodological differences exist. Observational studies typically rely on food frequency questionnaires (FFQs) to estimate dietary β‐carotene intake, which are prone to residual confounding from overall healthy dietary patterns, socioeconomic status, and lifestyle factors. In contrast, Mendelian randomization (MR) leverages genetic variants as proxies for lifelong β‐carotene exposure, inherently minimizing bias from confounding factors such as healthy user bias and reverse causality. Second, the outcome measures differ. While prior studies often focused on semen quality parameters—such as total motile sperm count or fertilization rate—our MR analysis used clinical infertility diagnosis as the outcome. Although related, these endpoints represent different levels of the biological hierarchy, and it is possible that MR lacked sufficient power to detect subtle effects limited to sperm parameters. Finally, differences in study populations should be considered. Observational studies often recruited specific subgroups—such as infertile patients or university students—who may have higher baseline oxidative stress levels and therefore be more responsive to antioxidant effects. In contrast, MR reflects average population‐level effects, potentially masking benefits among high‐risk subgroups. Taken together, the positive associations observed in observational studies may reflect the effects of carotenoid‐rich dietary patterns or synergistic actions of other antioxidants, rather than a direct causal effect of β‐carotene itself. As previously demonstrated, elevated vitamin C and β‐carotene levels in men are positively associated with fertilization rates among infertile couples, yet fail to translate into improved live‐birth outcomes (Li et al. 2019).

### 4.4 Phosphorus: Linking Metabolism to Male Reproductive Health

Phosphorus, the body's second‐most abundant mineral, is essential for bone formation, DNA synthesis, and energy metabolism. Serum phosphorus (2.5–4.5 mg/dL) is regulated by the bone–kidney–intestine network, involving PTH, fibroblast growth factor‐23 (FGF–23), Klotho, and vitamin D (Calvo and Lamberg‐Allardt 2015).

A negative correlation between serum phosphorus and male fertility has been supported by multiple lines of evidence. For example, in studies involving Wistar Kyoto rats and C57BL/6 mice, malondialdehyde (MDA) levels—a marker of oxidative stress—were significantly higher than that in the normal diet group. Elevated reactive oxygen species (ROS) can damage intracellular structures, induce apoptosis of germ cells, and impair spermatogenesis, ultimately leading to a reduced sperm count and motility (Suzuki et al. 2013). Our analysis aligns with these findings. Chronic high phosphorus intake imposed a burden of renal excretion, inducing chronic kidney disease (CKD), which, in calcium and phosphorus metabolism, inhibits testosterone synthesis, stimulates the secretion of parathyroid hormone, and interferes with the synthesis of reproductive hormones. In a CKD mouse model, high‐phosphorus diets have been shown to exacerbate testicular damage, which is consistent with the research results and suggests that clinicians should pay attention to the potential risks to reproductive function in relevant patients (Tsao et al. 2020). However, clinical data also found that the risk of erectile dysfunction in the group with the highest one‐third of phosphorus intake is 33% lower than that in the group with the lowest one‐third of phosphorus intake (OR = 0.67; 95% CI = 0.52–0.87; *p* = 0.0024), indicating that phosphorus is also an essential element in male reproduction (Deng et al. 2024).

### 4.5 Limitations and Suggestions for Future Research

While this Mendelian randomization study provides valuable causal insights into micronutrient–infertility relationships, several limitations warrant acknowledgment. First, our analysis was restricted to European‐ancestry populations, limiting the generalizability of findings to other ethnic groups with distinct genetic architectures, dietary patterns, and environmental contexts. Second, we relied on circulating micronutrient biomarkers as proxies for biologically available nutrient pools; these may not fully reflect tissue‐specific concentrations or long‐term nutritional status, particularly for nutrients with complex storage and regulation mechanisms. Third, though MR methods mitigate confounding, residual pleiotropy cannot be entirely excluded, and weak instrument bias may persist for nutrients with limited genome‐wide significant SNPs. Finally, the smaller male infertility sample size (680 cases vs. 6481 female cases) may have reduced power to detect modest associations.

Future research should prioritize large‐scale validation and mechanistic exploration to translate these genetic insights into clinical applications. First, multi‐ancestry Mendelian randomization and cohort studies are essential to verify the generalizability of findings beyond European populations, accounting for ethnic variations in micronutrient metabolism and infertility etiology. Second, tissue‐specific investigations—particularly quantifying micronutrient concentrations in ovarian follicles, testes, endometrium, and seminal fluid—would elucidate site‐specific mechanisms behind the observed sex‐dimorphic effects. Third, integrating gene–environment interaction analyses could reveal how dietary patterns or environmental exposures modify genetic effects on micronutrient bioavailability. Finally, randomized controlled trials targeting the implicated pathways—such as selenium/iron/β‐carotene supplementation in women with idiopathic infertility or dietary phosphorus reduction in subfertile men—are warranted to establish causal efficacy and optimal dosing regimens, ultimately bridging genetic epidemiology with precision nutrition interventions.

## 5 Conclusion

The findings of this study indicate that selenium significantly reduces the risk of female infertility, likely through its antioxidant properties, while showing no significant association with male infertility. Iron appears to enhance female reproductive outcomes by modulating ovarian bioenergetic metabolism. β‐Carotene is positively correlated with female fertility; in contrast to earlier findings, however, the present study indicates no significant association with male reproductive capacity. Elevated blood phosphorus levels may pose sex‐specific risks to male fertility by aggravating testicular oxidative stress and disrupting hormonal balance.

This study provides important evidence supporting sex‐specific nutritional interventions in reproductive health. It marks a shift from generalized dietary recommendations toward more precise, targeted strategies for the prevention and management of infertility.

## Author Contributions

**Jiaxin Zhang:** conceptualization (lead), data curation (lead), formal analysis (lead), investigation (lead), methodology (lead), project administration (lead), resources (lead), writing – original draft (lead). **Bo Hu:** data curation (equal), writing – review and editing (equal). **Tao Wang:** data curation (equal), supervision (equal), validation (equal). **Xiaotong Peng:** data curation (equal), visualization (equal). **Pusheng Yang:** conceptualization (equal), resources (equal). **Wenwen Liu:** funding acquisition (equal), writing – review and editing (equal). **Yaxin Miao:** software (equal). **Xin Lin:** data curation (equal). **Sun Jing:** study design (lead), writing‐review (lead).

## Ethics Statement

The data used in this paper is publicly available, ethically approved.

## Consent

The subjects have given informed consent.

## Conflicts of Interest

The authors declare no conflicts of interest.

## Supporting information


**Figures S1–S4:** fsn371084‐sup‐0001‐Figures.docx.

**Tables S1–S3:** fsn371084‐sup‐0002‐TableS1‐S3.docx.

## Data Availability

Data openly available in a public repository, those data can be found here https://gwas.mrcieu.ac.uk/.

## References

[fsn371084-bib-0001] Barchielli, G. , A. Capperucci , and D. Tanini . 2022. “The Role of Selenium in Pathologies: An Updated Review.” Antioxidants 11, no. 2: 251. 10.3390/antiox11020251. 35204134 PMC8868242

[fsn371084-bib-0002] Basini, G. , and C. Tamanini . 2000. “Selenium Stimulates Estradiol Production in Bovine Granulosa Cells: Possible Involvement of Nitric Oxide.” Domestic Animal Endocrinology 18, no. 1: 1–17. 10.1016/s0739-7240(99)00059-4. 10701760

[fsn371084-bib-0003] Benatta, M. , R. Kettache , N. Buchholz , and A. Trinchieri . 2020. “The Impact of Nutrition and Lifestyle on Male Fertility.” Archivio Italiano di Urologia, Andrologia 92, no. 2: 121. 10.4081/aiua.2020.2.121. 32597116

[fsn371084-bib-0004] Briggs, D. A. , D. J. Sharp , D. Miller , and R. G. Gosden . 1999. “Transferrin in the Developing Ovarian Follicle: Evidence for De‐Novo Expression by Granulosa Cells.” Molecular Human Reproduction 5, no. 12: 1107–1114. 10.1093/molehr/5.12.1107. 10587364

[fsn371084-bib-0005] Brooke, V. R. , B. Leah Hawkins , C. Katharine F , L. Shane , H. Mark D , and M. Stacey A . 2017. “Lifestyle and In Vitro Fertilization: What Do Patients Believe?” Fertility Research and Practice 2: 5. 10.1186/s40738-016-0026-5. PMC5424337 28620538

[fsn371084-bib-0006] Calvo, M. S. , and C. J. Lamberg‐Allardt . 2015. “Phosphorus.” Advances in Nutrition 6, no. 6: 860–862. 10.3945/an.115.008516. 26567206 PMC4642415

[fsn371084-bib-0007] Chavarro, J. E. , J. W. Rich‐Edwards , B. A. Rosner , and W. C. Willett . 2006. “Iron Intake and Risk of Ovulatory Infertility.” Obstetrics and Gynecology 108, no. 5: 1145–1152. 10.1097/01.AOG.0000238333.37423.ab. 17077236

[fsn371084-bib-0008] De Cosmi, V. , F. Parazzini , C. Agostoni , et al. 2021. “Antioxidant Vitamins and Carotenoids Intake and the Association With Poor Semen Quality: A Cross‐Sectional Analysis of Men Referring to an Italian Fertility Clinic.” Frontiers in Nutrition 8: 737077. 10.3389/fnut.2021.737077. 34671631 PMC8520935

[fsn371084-bib-0009] Deng, C. Y. , X. P. Ke , and X. G. Guo . 2024. “Dietary Calcium, Phosphorus, and Potassium Intake Associated With Erectile Dysfunction in the National Health and Nutrition Examination Survey (NHANES) 2001 to 2004.” PLoS One 19, no. 2: e0297129. 10.1371/journal.pone.0297129. 38381721 PMC10880986

[fsn371084-bib-0010] Duntas, L. H. 2020. “Selenium and At‐Risk Pregnancy: Challenges and Controversies.” Thyroid Research 13: 16. 10.1186/s13044-020-00090-x. 33014140 PMC7528225

[fsn371084-bib-0011] Ferrucci, L. , J. R. Perry , A. Matteini , et al. 2009. “Common Variation in the Beta‐Carotene 15,15′‐Monooxygenase 1 Gene Affects Circulating Levels of Carotenoids: A Genome‐Wide Association Study.” American Journal of Human Genetics 84, no. 2: 123–133. 10.1016/j.ajhg.2008.12.019. 19185284 PMC2668002

[fsn371084-bib-0012] Gärtner, R. , B. C. Gasnier , J. W. Dietrich , B. Krebs , and M. W. Angstwurm . 2002. “Selenium Supplementation in Patients With Autoimmune Thyroiditis Decreases Thyroid Peroxidase Antibodies Concentrations.” Journal of Clinical Endocrinology and Metabolism 87, no. 4: 1687–1691. 10.1210/jcem.87.4.8421. 11932302

[fsn371084-bib-0013] Georgsen, M. , M. C. Krog , A. S. Korsholm , et al. 2021. “Serum Ferritin Level Is Inversely Related to Number of Previous Pregnancy Losses in Women With Recurrent Pregnancy Loss.” Fertility and Sterility 115, no. 2: 389–396. 10.1016/j.fertnstert.2020.08.1410. 32988613

[fsn371084-bib-0014] Gonzalez‐Martin, R. , A. Palomar , S. Perez‐Deben , et al. 2024. “Higher Concentrations of Essential Trace Elements in Women Undergoing IVF May Be Associated With Poor Reproductive Outcomes Following Single Euploid Embryo Transfer.” Cells 13, no. 10: 839. 10.3390/cells13100839. 38786061 PMC11119764

[fsn371084-bib-0015] Grarup, N. , P. Sulem , C. H. Sandholt , et al. 2013. “Genetic Architecture of Vitamin B12 and Folate Levels Uncovered Applying Deeply Sequenced Large Datasets.” PLoS Genetics 9, no. 6: e1003530. 10.1371/journal.pgen.1003530. 23754956 PMC3674994

[fsn371084-bib-0016] Hauser, R. , A. J. Gaskins , I. Souter , et al. 2016. “Urinary Phthalate Metabolite Concentrations and Reproductive Outcomes Among Women Undergoing In Vitro Fertilization: Results From the EARTH Study.” Environmental Health Perspectives 124, no. 6: 831–839. 10.1289/ehp.1509760. 26545148 PMC4892919

[fsn371084-bib-0017] Hawkes, W. C. , Z. Alkan , and K. Wong . 2009. “Selenium Supplementation Does Not Affect Testicular Selenium Status or Semen Quality in North American Men.” Journal of Andrology 30, no. 5: 525–533. 10.2164/jandrol.108.006940. 19342701

[fsn371084-bib-0018] Hofstee, P. , L. A. Bartho , D. R. McKeating , et al. 2019. “Maternal Selenium Deficiency During Pregnancy in Mice Increases Thyroid Hormone Concentrations, Alters Placental Function and Reduces Fetal Growth.” Journal of Physiology 597, no. 23: 5597–5617. 10.1113/jp278473. 31562642

[fsn371084-bib-0019] Huang, S. A. , D. M. Dorfman , D. R. Genest , D. Salvatore , and P. R. Larsen . 2003. “Type 3 Iodothyronine Deiodinase Is Highly Expressed in the Human Uteroplacental Unit and in Fetal Epithelium.” Journal of Clinical Endocrinology and Metabolism 88, no. 3: 1384–1388. 10.1210/jc.2002-021291. 12629133

[fsn371084-bib-0020] Hummitzsch, K. , J. E. Kelly , N. Hatzirodos , et al. 2024. “Expression Levels of the Selenium‐Uptake Receptor LRP8, the Antioxidant Selenoprotein GPX1 and Steroidogenic Enzymes Correlate in Granulosa Cells.” Reproduction and Fertility 5, no. 3: 74. 10.1530/raf-23-0074. PMC11301534 38990713

[fsn371084-bib-0021] Kestenbaum, B. , N. L. Glazer , A. Köttgen , et al. 2010. “Common Genetic Variants Associate With Serum Phosphorus Concentration.” Journal of the American Society of Nephrology 21, no. 7: 1223–1232. 10.1681/asn.2009111104. 20558539 PMC3152230

[fsn371084-bib-0022] Kim, K. , E. F. Schisterman , R. M. Silver , et al. 2018. “Shorter Time to Pregnancy With Increasing Preconception Carotene Concentrations Among Women With 1–2 Previous Pregnancy Losses.” American Journal of Epidemiology 187, no. 9: 1907–1915. 10.1093/aje/kwy101. 29767694 PMC6118073

[fsn371084-bib-0023] Lakoma, K. , O. Kukharuk , and D. Sliz . 2023. “The Influence of Metabolic Factors and Diet on Fertility.” Nutrients 15, no. 5: 1180. 10.3390/nu15051180. 36904180 PMC10005661

[fsn371084-bib-0024] Li, M. C. , Y. H. Chiu , A. J. Gaskins , et al. 2019. “Men's Intake of Vitamin C and β‐Carotene Is Positively Related to Fertilization Rate but Not to Live Birth Rate in Couples Undergoing Infertility Treatment.” Journal of Nutrition 149, no. 11: 1977–1984. 10.1093/jn/nxz149. 31287143 PMC6825820

[fsn371084-bib-0025] Major, J. M. , K. Yu , W. Wheeler , et al. 2011. “Genome‐Wide Association Study Identifies Common Variants Associated With Circulating Vitamin E Levels.” Human Molecular Genetics 20, no. 19: 3876–3883. 10.1093/hmg/ddr296. 21729881 PMC3168288

[fsn371084-bib-0026] Mélodie, V. B. , and W. Christine . 2018. “Fertility and Infertility: Definition and Epidemiology.” Clinical Biochemistry 62: 12. 10.1016/j.clinbiochem.2018.03.012. 29555319

[fsn371084-bib-0027] Mesdaghinia, E. , A. Rahavi , F. Bahmani , N. Sharifi , and Z. Asemi . 2017. “Clinical and Metabolic Response to Selenium Supplementation in Pregnant Women at Risk for Intrauterine Growth Restriction: Randomized, Double‐Blind, Placebo‐Controlled Trial.” Biological Trace Element Research 178, no. 1: 14–21. 10.1007/s12011-016-0911-0. 27928721

[fsn371084-bib-0028] Meyer, T. E. , G. C. Verwoert , S. J. Hwang , et al. 2010. “Genome‐Wide Association Studies of Serum Magnesium, Potassium, and Sodium Concentrations Identify Six Loci Influencing Serum Magnesium Levels.” PLoS Genetics 6, no. 8: 1001045. 10.1371/journal.pgen.1001045. PMC2916845 20700443

[fsn371084-bib-0029] Mínguez‐Alarcón, L. , J. Mendiola , J. J. López‐Espín , et al. 2012. “Dietary Intake of Antioxidant Nutrients Is Associated With Semen Quality in Young University Students.” Human Reproduction 27, no. 9: 2807–2814. 10.1093/humrep/des247. 22752607

[fsn371084-bib-0030] Mojadadi, A. , A. Au , W. Salah , P. Witting , and G. Ahmad . 2021. “Role for Selenium in Metabolic Homeostasis and Human Reproduction.” Nutrients 13, no. 9: 3256. 10.3390/nu13093256. 34579133 PMC8469766

[fsn371084-bib-0031] Mondul, A. M. , K. Yu , W. Wheeler , et al. 2011. “Genome‐Wide Association Study of Circulating Retinol Levels.” Human Molecular Genetics 20, no. 23: 4724–4731. 10.1093/hmg/ddr387. 21878437 PMC3209826

[fsn371084-bib-0032] O'Seaghdha, C. M. , H. Wu , Q. Yang , et al. 2013. “Meta‐Analysis of Genome‐Wide Association Studies Identifies Six New Loci for Serum Calcium Concentrations.” PLoS Genetics 9, no. 9: e1003796. 10.1371/journal.pgen.1003796. 24068962 PMC3778004

[fsn371084-bib-0033] Polyzos, N. P. , E. Anckaert , L. Guzman , et al. 2014. “Vitamin D Deficiency and Pregnancy Rates in Women Undergoing Single Embryo, Blastocyst Stage, Transfer (SET) for IVF/ICSI.” Human Reproduction 29, no. 9: 2032–2040. 10.1093/humrep/deu156. 24951484

[fsn371084-bib-0034] Robert, D. N. 2006. “International Disparities in Access to Infertility Services.” Fertility and Sterility 85, no. 4: 66. 10.1016/j.fertnstert.2005.08.066. 16580367

[fsn371084-bib-0035] Ruder, E. H. , T. J. Hartman , R. H. Reindollar , and M. B. Goldman . 2014. “Female Dietary Antioxidant Intake and Time to Pregnancy Among Couples Treated for Unexplained Infertility.” Fertility and Sterility 101, no. 3: 759–766. 10.1016/j.fertnstert.2013.11.008. 24355050 PMC3943921

[fsn371084-bib-0036] Rushton, D. H. , I. D. Ramsay , J. J. Gilkes , and M. J. Norris . 1991. “Ferritin and Fertility.” Lancet 337, no. 8756: 1554. 10.1016/0140-6736(91)93255-8. 1675411

[fsn371084-bib-0037] Safarinejad, M. R. , and S. Safarinejad . 2009. “Efficacy of Selenium and/or N‐Acetyl‐Cysteine for Improving Semen Parameters in Infertile Men: A Double‐Blind, Placebo Controlled, Randomized Study.” Journal of Urology 181, no. 2: 741–751. 10.1016/j.juro.2008.10.015. 19091331

[fsn371084-bib-0038] Safiyeh, F. D. , M. Mojgan , S. Parviz , M. A. Sakineh , and S. O. Behnaz . 2021. “The Effect of Selenium and Vitamin E Supplementation on Anti‐Mullerian Hormone and Antral Follicle Count in Infertile Women With Occult Premature Ovarian Insufficiency: A Randomized Controlled Clinical Trial.” Complementary Therapies in Medicine 56: 102533. 10.1016/j.ctim.2020.102533. 33197657

[fsn371084-bib-0039] Salas‐Huetos, A. , M. Bulló , and J. Salas‐Salvadó . 2017. “Dietary Patterns, Foods and Nutrients in Male Fertility Parameters and Fecundability: A Systematic Review of Observational Studies.” Human Reproduction Update 23, no. 4: 371–389. 10.1093/humupd/dmx006. 28333357

[fsn371084-bib-0040] Schomburg, L. 2016. “Sex‐Specific Differences in Biological Effects and Metabolism of Selenium.” In Its Molecular Biology and Role in Human Health, 377–388. Springer International Publishing.

[fsn371084-bib-0041] Seale, L. A. , A. N. Ogawa‐Wong , and M. J. Berry . 2018. “Sexual Dimorphism in Selenium Metabolism and Selenoproteins.” Free Radical Biology & Medicine 127: 198–205. 10.1016/j.freeradbiomed.2018.03.036. 29572096 PMC6150850

[fsn371084-bib-0042] Showell, M. G. , R. Mackenzie‐Proctor , V. Jordan , and R. J. Hart . 2020. “Antioxidants for Female Subfertility.” Cochrane Database of Systematic Reviews 8, no. 8: Cd007807. 10.1002/14651858.CD007807.pub4. 32851663 PMC8094745

[fsn371084-bib-0043] Simpson, S. , and L. Pal . 2023. “Vitamin D and Infertility.” Current Opinion in Obstetrics & Gynecology 35, no. 4: 300–305. 10.1097/gco.0000000000000887. 37266579

[fsn371084-bib-0044] Skoracka, K. , P. Eder , L. Łykowska‐Szuber , A. Dobrowolska , and I. Krela‐Kaźmierczak . 2020. “Diet and Nutritional Factors in Male (In)fertility‐Underestimated Factors.” Journal of Clinical Medicine 9, no. 5: 1400. 10.3390/jcm9051400. 32397485 PMC7291266

[fsn371084-bib-0045] Suks, M. , B. Carlo , B. Luca , et al. 2021. “European Association of Urology Guidelines on Male Sexual and Reproductive Health: 2021 Update on Male Infertility.” European Urology 80, no. 5: 14. 10.1016/j.eururo.2021.08.014. 34511305

[fsn371084-bib-0046] Sulagna, D. , G. Bapi , C. Hira , R. Shubhadeep , and S. Pallav . 2021. “Environmental and Occupational Exposure of Metals and Female Reproductive Health.” Environmental Science and Pollution Research International 29, no. 41: 9. 10.1007/s11356-021-16581-9. 34558053

[fsn371084-bib-0047] Suzuki, Y. , G. Ichihara , S. M. Sahabudeen , et al. 2013. “Rats With Metabolic Syndrome Resist the Protective Effects of N‐Acetyl l‐Cystein Against Impaired Spermatogenesis Induced by High‐Phosphorus/Zinc‐Free Diet.” Experimental and Toxicologic Pathology 65, no. 7–8: 1173–1182. 10.1016/j.etp.2013.05.009. 23810784

[fsn371084-bib-0048] Tanaka, T. , P. Scheet , B. Giusti , et al. 2009. “Genome‐Wide Association Study of Vitamin B6, Vitamin B12, Folate, and Homocysteine Blood Concentrations.” American Journal of Human Genetics 84, no. 4: 477–482. 10.1016/j.ajhg.2009.02.011. 19303062 PMC2667971

[fsn371084-bib-0049] Tonai, S. , A. Kawabata , T. Nakanishi , et al. 2020. “Iron Deficiency Induces Female Infertile in Order to Failure of Follicular Development in Mice.” Journal of Reproduction and Development 66, no. 5: 475–483. 10.1262/jrd.2020-074. 32713881 PMC7593635

[fsn371084-bib-0050] Tsao, C. W. , Y. J. Hsu , T. C. Chang , S. T. Wu , T. L. Cha , and C. Y. Liu . 2020. “A High Phosphorus Diet Impairs Testicular Function and Spermatogenesis in Male Mice With Chronic Kidney Disease.” Nutrients 12, no. 9: 2624. 10.3390/nu12092624. 32872125 PMC7551469

[fsn371084-bib-0051] Uhm, S. J. , M. K. Gupta , J. H. Yang , S. H. Lee , and H. T. Lee . 2007. “Selenium Improves the Developmental Ability and Reduces the Apoptosis in Porcine Parthenotes.” Molecular Reproduction and Development 74, no. 11: 1386–1394. 10.1002/mrd.20701. 17342738

[fsn371084-bib-0052] Ursini, F. , S. Heim , M. Kiess , et al. 1999. “Dual Function of the Selenoprotein PHGPx During Sperm Maturation.” Science 285, no. 5432: 1393–1396. 10.1126/science.285.5432.1393. 10464096

[fsn371084-bib-0053] Yu, S. , Y. Zhao , Y. Feng , et al. 2019. “β‐Carotene Improves Oocyte Development and Maturation Under Oxidative Stress In Vitro.” In Vitro Cellular & Developmental Biology. Animal 55, no. 7: 548–558. 10.1007/s11626-019-00373-0. 31313007

[fsn371084-bib-0054] Zaixiang, T. , W. Ying , X. Xing , S. Ziyuan , and S. Wei . 2024. “Socioeconomic Status, Individual Behaviors and Risk for Lymphomas: A Mendelian Randomization Study.” Journal of Cancer 15, no. 12: 96413. 10.7150/jca.96413. PMC11190779 38911370

