# Supplementary material for: Causal Associations of Circulating Micronutrients With the Risk of Infertility: A Mendelian Randomization Study
Source: Food Sci Nutr. 2025 Oct 28;13(11):e71084. doi: 10.1002/fsn3.71084 (PMC12567636; doi:10.1002/fsn3.71084)
Supplement: Supplementary file 1 — Figures S1–S4: fsn371084‐sup‐0001‐Figures.docx. [file FSN3-13-e71084-s002.docx]

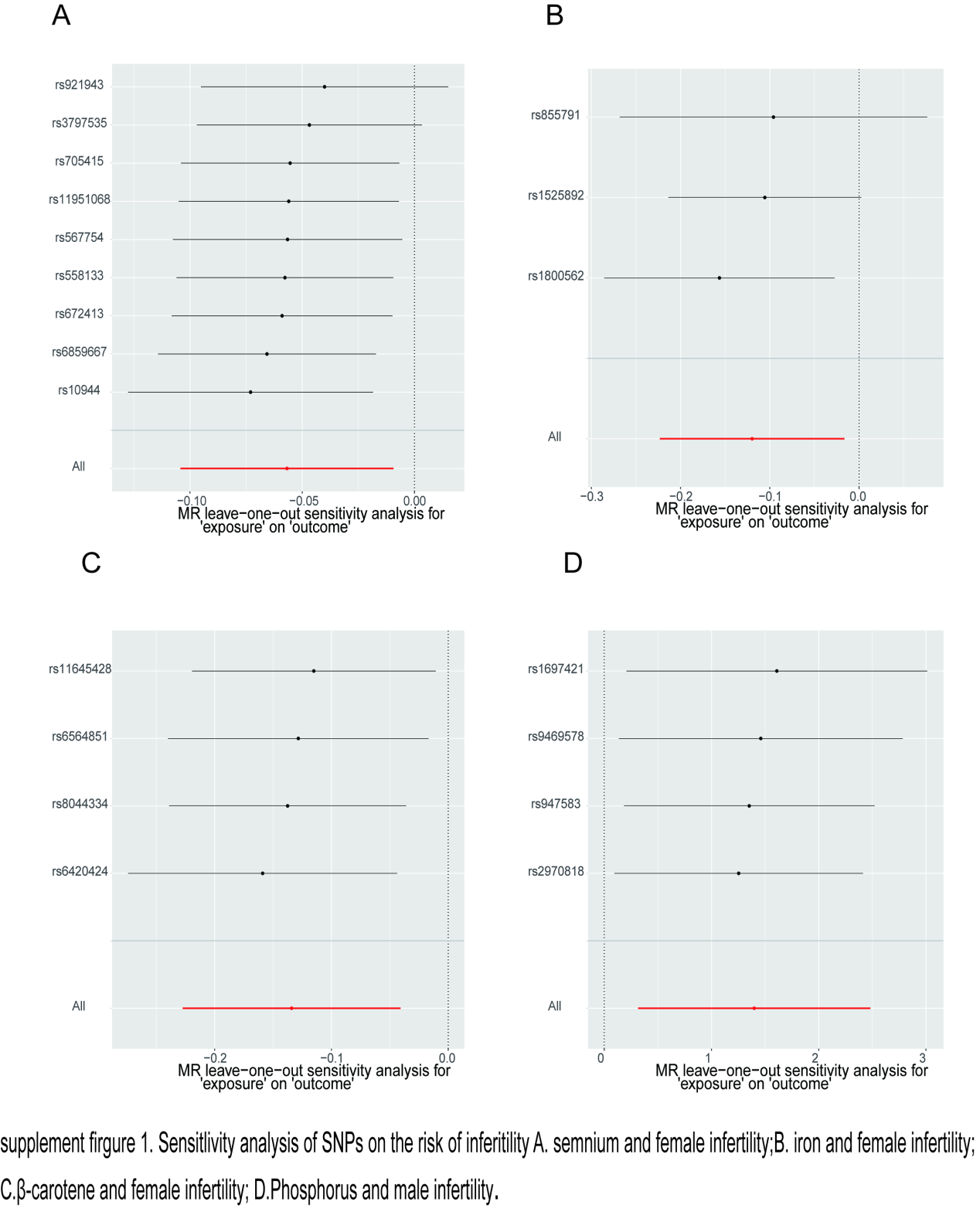
Supplement Firgure 1. Sensitlivity analysis of SNPs on the risk of inferitility A. semnium and female infertility;B. iron and female infertility; C.β-carotene and female infertility; D.Phosphorus and male infertility.


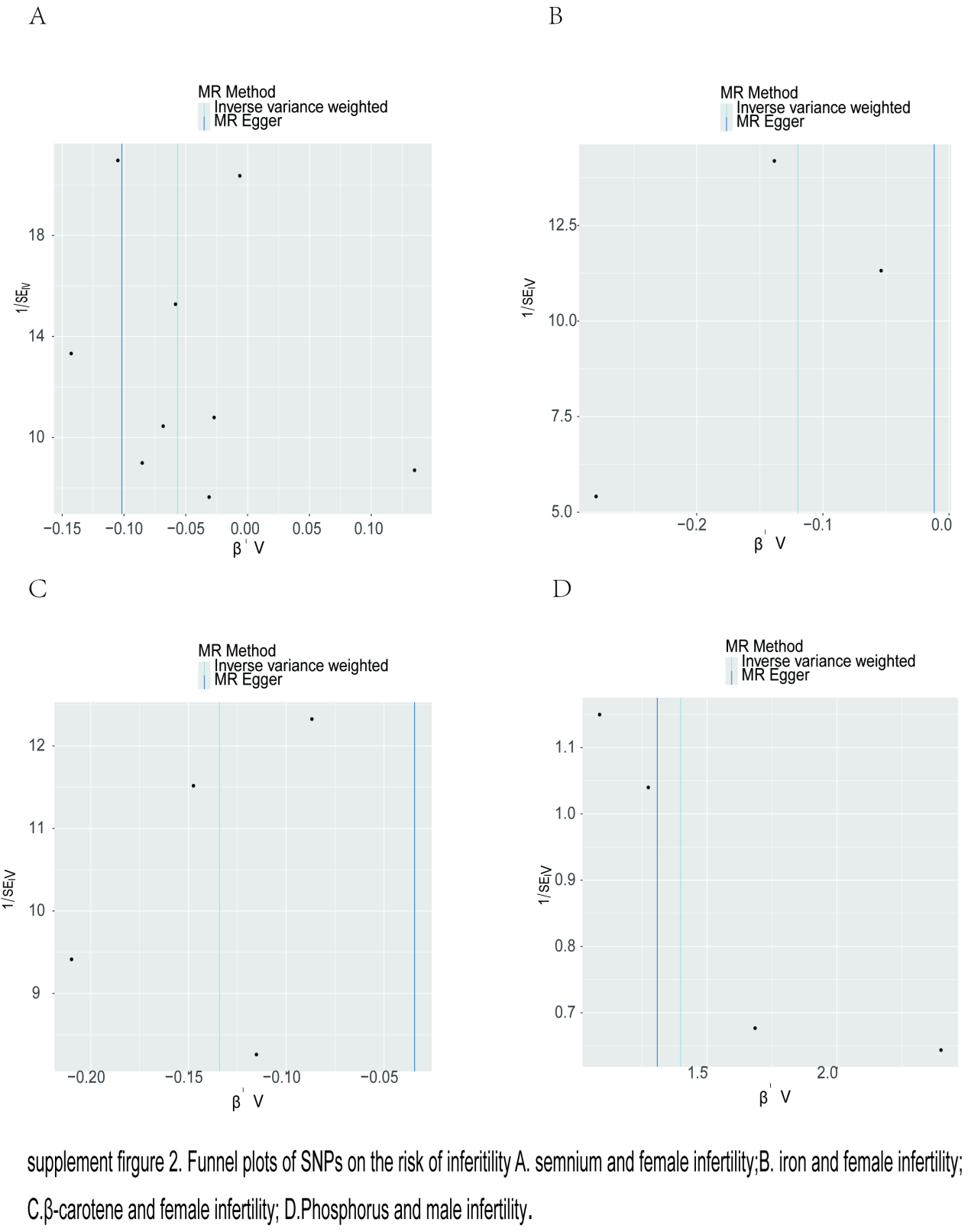
Supplement Firgure 2. Funnel plots of SNPs on the risk of inferitility A. semnium and female infertility;B. iron and female infertility; C.β-carotene and female infertility; D.Phosphorus and male infertility.


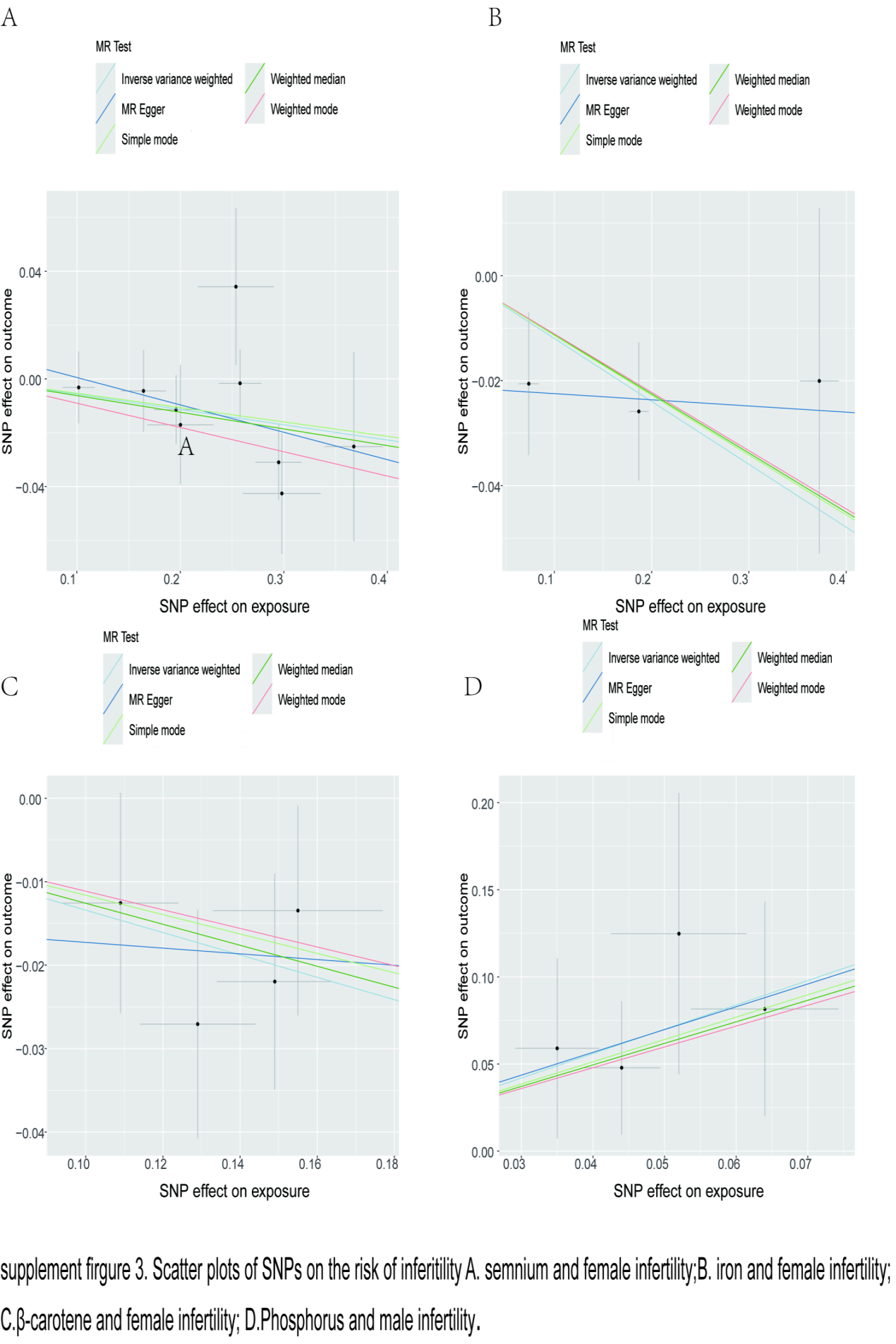


Supplement Firgure 3. Scatter plots of SNPs on the risk of inferitility A. semnium and female infertility;B. iron and female infertility;

C.β-carotene and female infertility; D.Phosphorus and male infertility

.


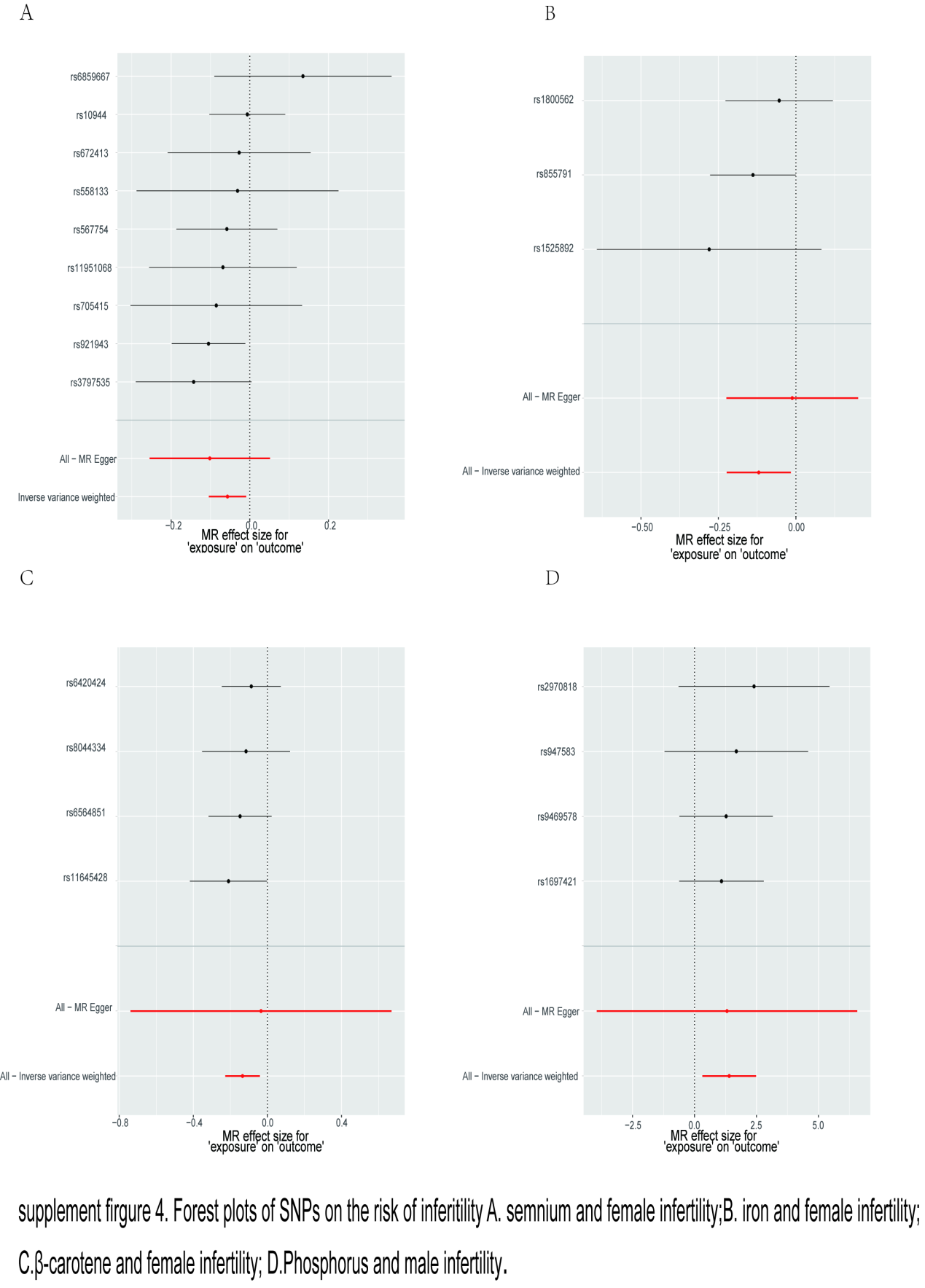
Supplement Firgure 4. Forest plots of SNPs on the risk of inferitility A. semnium and female infertility;B. iron and female infertility;

C.β-carotene and female infertility; D.Phosphorus and male infertility.
