# Supplementary material for: Causal Associations of Circulating Micronutrients With the Risk of Infertility: A Mendelian Randomization Study
Source: Food Sci Nutr. 2025 Oct 28;13(11):e71084. doi: 10.1002/fsn3.71084 (PMC12567636; doi:10.1002/fsn3.71084)
Supplement: Supplementary file 2 — Tables S1–S3: fsn371084‐sup‐0002‐TableS1‐S3.docx. [file FSN3-13-e71084-s001.docx]

| Supplementary Table 1. Summary for proxy for missing SNPs in outcome | | | | | | | | | |
| --- | --- | --- | --- | --- | --- | --- | --- | --- | --- |
| Sentinel | Proxy | Chr. | Proxy Pos. | Distance (bp) | LD r² | LD D | LD D' | Proxy Allele B | Allele B Frequency |
| rs1789953 | rs1788466 | 21 | 44483773 | 837 | 0.82 | 0.13 | 0.99 | A | 0.189 |
| rs6586282 | rs6586281 | 21 | 44478393 | -104 | 1 | 0.13 | 1 | A | 0.153 |
| rs6586282 | rs6586283 | 21 | 44478680 | 183 | 1 | 0.13 | 1 | C | 0.153 |

| Supplementary Table 2. MR analysis estimates for the association of micronugtrients with risk of female infertility | | | | | | | | | | |
| --- | --- | --- | --- | --- | --- | --- | --- | --- | --- | --- |
| name | method | nsnp | b | se | pval | lo_ci | up_ci | or | or_lci95 | or_uci95 |
| vit A | Inverse variance weighted | 2 | 0.118 | 0.432 | 0.784 | -0.728 | 0.964 | 1.125 | 0.483 | 2.623 |
| vit E | Inverse variance weighted | 3 | 0.208 | 0.297 | 0.484 | -0.374 | 0.790 | 1.231 | 0.688 | 2.204 |
| vit E | MR Egger | 3 | -0.334 | 2.146 | 0.902 | -4.541 | 3.873 | 0.716 | 0.011 | 48.076 |
| vit E | Weighted median | 3 | 0.210 | 0.359 | 0.558 | -0.492 | 0.913 | 1.234 | 0.611 | 2.492 |
| vit E | Weighted mode | 3 | 0.157 | 0.420 | 0.745 | -0.667 | 0.981 | 1.170 | 0.513 | 2.666 |
| vit E | Simple mode | 3 | 0.252 | 0.410 | 0.601 | -0.551 | 1.055 | 1.287 | 0.576 | 2.873 |
| vit D | Inverse variance weighted | 6 | -0.040 | 0.046 | 0.385 | -0.131 | 0.050 | 0.961 | 0.878 | 1.052 |
| vit D | MR Egger | 6 | -0.003 | 0.084 | 0.972 | -0.168 | 0.162 | 0.997 | 0.846 | 1.175 |
| vit D | Weighted median | 6 | -0.041 | 0.052 | 0.431 | -0.142 | 0.061 | 0.960 | 0.868 | 1.062 |
| vit D | Weighted mode | 6 | -0.040 | 0.053 | 0.480 | -0.144 | 0.063 | 0.961 | 0.866 | 1.065 |
| vit D | Simple mode | 6 | -0.035 | 0.087 | 0.702 | -0.207 | 0.136 | 0.965 | 0.813 | 1.146 |
| vit B6 | Inverse variance weighted | 2 | 0.068 | 0.084 | 0.413 | -0.095 | 0.232 | 1.071 | 0.909 | 1.261 |
| vit B12 | Inverse variance weighted | 10 | 0.021 | 0.039 | 0.585 | -0.055 | 0.098 | 1.022 | 0.946 | 1.103 |
| vit B12 | MR Egger | 10 | -0.036 | 0.061 | 0.568 | -0.155 | 0.083 | 0.965 | 0.856 | 1.086 |
| vit B12 | Weighted median | 10 | 0.036 | 0.040 | 0.370 | -0.042 | 0.114 | 1.036 | 0.959 | 1.120 |
| vit B12 | Weighted mode | 10 | 0.042 | 0.039 | 0.308 | -0.034 | 0.118 | 1.043 | 0.966 | 1.125 |
| vit B12 | Simple mode | 10 | 0.042 | 0.073 | 0.578 | -0.101 | 0.184 | 1.043 | 0.904 | 1.202 |
| vit C | Inverse variance weighted | 10 | 0.123 | 0.072 | 0.086 | -0.018 | 0.263 | 1.131 | 0.983 | 1.301 |
| vit C | MR Egger | 10 | 0.057 | 0.124 | 0.657 | -0.186 | 0.300 | 1.059 | 0.831 | 1.350 |
| vit C | Weighted median | 10 | 0.133 | 0.093 | 0.153 | -0.049 | 0.315 | 1.142 | 0.952 | 1.370 |
| vit C | Weighted mode | 10 | 0.130 | 0.112 | 0.277 | -0.090 | 0.349 | 1.138 | 0.914 | 1.418 |
| vit C | Simple mode | 10 | 0.130 | 0.154 | 0.423 | -0.173 | 0.432 | 1.138 | 0.841 | 1.540 |
| Mg | Inverse variance weighted | 5 | 0.076 | 0.976 | 0.938 | -1.837 | 1.990 | 1.079 | 0.159 | 7.313 |
| Mg | MR Egger | 5 | -0.745 | 3.140 | 0.828 | -6.899 | 5.409 | 0.475 | 0.001 | 223.318 |
| Mg | Weighted median | 5 | -0.190 | 1.060 | 0.858 | -2.268 | 1.888 | 0.827 | 0.104 | 6.606 |
| Mg | Weighted mode | 5 | -0.135 | 1.186 | 0.915 | -2.460 | 2.190 | 0.874 | 0.085 | 8.937 |
| Mg | Simple mode | 5 | -1.631 | 1.557 | 0.354 | -4.682 | 1.420 | 0.196 | 0.009 | 4.137 |
| se | Inverse variance weighted | 9 | -0.057 | 0.024 | 0.019 | -0.104 | -0.009 | 0.945 | 0.901 | 0.991 |
| se | MR Egger | 9 | -0.102 | 0.078 | 0.234 | -0.255 | 0.051 | 0.903 | 0.775 | 1.053 |
| se | Weighted median | 9 | -0.062 | 0.031 | 0.049 | -0.124 | 0.000 | 0.940 | 0.884 | 1.000 |
| se | Weighted mode | 9 | -0.090 | 0.045 | 0.079 | -0.178 | -0.002 | 0.914 | 0.837 | 0.998 |
| se | Simple mode | 9 | -0.053 | 0.048 | 0.303 | -0.148 | 0.041 | 0.948 | 0.862 | 1.042 |
| Folate | Inverse variance weighted | 2 | -0.199 | 0.141 | 0.157 | -0.474 | 0.076 | 0.820 | 0.622 | 1.079 |
| Fe | Inverse variance weighted | 3 | -0.120 | 0.053 | 0.023 | -0.223 | -0.016 | 0.887 | 0.800 | 0.984 |
| Fe | MR Egger | 3 | -0.012 | 0.108 | 0.931 | -0.224 | 0.201 | 0.988 | 0.799 | 1.222 |
| Fe | Weighted median | 3 | -0.113 | 0.056 | 0.046 | -0.223 | -0.002 | 0.893 | 0.800 | 0.998 |
| Fe | Weighted mode | 3 | -0.111 | 0.066 | 0.234 | -0.240 | 0.018 | 0.895 | 0.786 | 1.018 |
| Fe | Simple mode | 3 | -0.114 | 0.072 | 0.255 | -0.255 | 0.028 | 0.892 | 0.775 | 1.028 |
| P | Inverse variance weighted | 4 | -0.032 | 0.181 | 0.860 | -0.386 | 0.322 | 0.969 | 0.680 | 1.380 |
| P | MR Egger | 4 | 0.070 | 0.874 | 0.943 | -1.643 | 1.784 | 1.073 | 0.193 | 5.953 |
| P | Weighted median | 4 | -0.039 | 0.207 | 0.849 | -0.445 | 0.366 | 0.961 | 0.641 | 1.442 |
| P | Weighted mode | 4 | -0.211 | 0.291 | 0.521 | -0.780 | 0.359 | 0.810 | 0.458 | 1.432 |
| P | Simple mode | 4 | -0.249 | 0.321 | 0.494 | -0.878 | 0.379 | 0.779 | 0.416 | 1.461 |
| β-carotene | Inverse variance weighted | 4 | -0.134 | 0.048 | 0.005 | -0.227 | -0.041 | 0.874 | 0.797 | 0.960 |
| β-carotene | MR Egger | 4 | -0.034 | 0.359 | 0.933 | -0.739 | 0.670 | 0.966 | 0.478 | 1.955 |
| β-carotene | Weighted median | 4 | -0.126 | 0.058 | 0.031 | -0.240 | -0.012 | 0.882 | 0.787 | 0.988 |
| β-carotene | Weighted mode | 4 | -0.111 | 0.071 | 0.216 | -0.251 | 0.028 | 0.895 | 0.778 | 1.029 |
| β-carotene | Simple mode | 4 | -0.116 | 0.070 | 0.196 | -0.254 | 0.021 | 0.890 | 0.776 | 1.022 |
| cu | Inverse variance weighted | 2 | -0.013 | 0.043 | 0.756 | -0.097 | 0.070 | 0.987 | 0.908 | 1.073 |
| zn | Inverse variance weighted | 2 | -0.005 | 0.063 | 0.932 | -0.129 | 0.118 | 0.995 | 0.879 | 1.126 |
| ca | Inverse variance weighted | 7 | 0.155 | 0.278 | 0.578 | -0.390 | 0.700 | 1.168 | 0.677 | 2.014 |
| ca | MR Egger | 7 | 0.119 | 0.565 | 0.842 | -0.988 | 1.226 | 1.126 | 0.372 | 3.407 |
| ca | Weighted median | 7 | 0.182 | 0.240 | 0.450 | -0.289 | 0.653 | 1.199 | 0.749 | 1.921 |
| ca | Weighted mode | 7 | 0.171 | 0.256 | 0.530 | -0.331 | 0.673 | 1.186 | 0.718 | 1.959 |
| ca | Simple mode | 7 | 0.091 | 0.389 | 0.823 | -0.672 | 0.853 | 1.095 | 0.511 | 2.348 |

| Supplementary Table 3. MR analysis estimates for the association of micronugtrients with risk of male infertility | | | | | | | | | | |
| --- | --- | --- | --- | --- | --- | --- | --- | --- | --- | --- |
|  | method | nsnp | b | se | pval | lo_ci | up_ci | or | or_lci95 | or_uci95 |
| vit A | Inverse variance weighted | 2 | -0.445 | 1.442 | 0.757 | -3.272 | 2.381 | 0.641 | 0.038 | 10.817 |
| vit E | Inverse variance weighted | 3 | 0.148 | 0.903 | 0.870 | -1.622 | 1.919 | 1.160 | 0.197 | 6.813 |
| vit E | MR Egger | 3 | -6.781 | 6.541 | 0.489 | -19.602 | 6.039 | 0.001 | 0.000 | 419.559 |
| vit E | Weighted median | 3 | -0.214 | 1.221 | 0.861 | -2.607 | 2.178 | 0.807 | 0.074 | 8.831 |
| vit E | Weighted mode | 3 | -0.455 | 1.338 | 0.766 | -3.078 | 2.167 | 0.634 | 0.046 | 8.734 |
| vit E | Simple mode | 3 | -0.282 | 1.455 | 0.864 | -3.132 | 2.569 | 0.755 | 0.044 | 13.055 |
| vit D | Inverse variance weighted | 6 | -0.226 | 0.200 | 0.259 | -0.618 | 0.166 | 0.798 | 0.539 | 1.181 |
| vit D | MR Egger | 6 | -0.418 | 0.391 | 0.345 | -1.184 | 0.348 | 0.658 | 0.306 | 1.416 |
| vit D | Weighted median | 6 | -0.248 | 0.156 | 0.111 | -0.552 | 0.057 | 0.781 | 0.576 | 1.059 |
| vit D | Weighted mode | 6 | -0.238 | 0.158 | 0.192 | -0.548 | 0.071 | 0.788 | 0.578 | 1.074 |
| vit D | Simple mode | 6 | -0.093 | 0.253 | 0.727 | -0.589 | 0.402 | 0.911 | 0.555 | 1.495 |
| vit B6 | Inverse variance weighted | 2 | 0.041 | 0.299 | 0.891 | -0.546 | 0.628 | 1.042 | 0.580 | 1.874 |
| vit B12 | Inverse variance weighted | 10 | -0.181 | 0.093 | 0.053 | -0.364 | 0.002 | 0.835 | 0.695 | 1.002 |
| vit B12 | MR Egger | 10 | -0.208 | 0.158 | 0.223 | -0.517 | 0.101 | 0.812 | 0.596 | 1.106 |
| vit B12 | Weighted median | 10 | -0.250 | 0.126 | 0.048 | -0.497 | -0.002 | 0.779 | 0.608 | 0.998 |
| vit B12 | Weighted mode | 10 | -0.238 | 0.137 | 0.116 | -0.506 | 0.030 | 0.788 | 0.603 | 1.030 |
| vit B12 | Simple mode | 10 | -0.202 | 0.216 | 0.375 | -0.626 | 0.222 | 0.817 | 0.535 | 1.249 |
| vit C | Inverse variance weighted | 10 | -0.324 | 0.217 | 0.135 | -0.748 | 0.101 | 0.724 | 0.473 | 1.106 |
| vit C | MR Egger | 10 | 0.172 | 0.373 | 0.656 | -0.559 | 0.904 | 1.188 | 0.572 | 2.469 |
| vit C | Weighted median | 10 | -0.419 | 0.287 | 0.145 | -0.982 | 0.144 | 0.658 | 0.375 | 1.155 |
| vit C | Weighted mode | 10 | 0.137 | 0.332 | 0.689 | -0.514 | 0.789 | 1.147 | 0.598 | 2.200 |
| vit C | Simple mode | 10 | -0.802 | 0.555 | 0.182 | -1.891 | 0.286 | 0.448 | 0.151 | 1.331 |
| Mg | Inverse variance weighted | 5 | -1.123 | 2.682 | 0.675 | -6.379 | 4.133 | 0.325 | 0.002 | 62.361 |
| Mg | MR Egger | 5 | -1.412 | 7.568 | 0.864 | -16.245 | 13.420 | 0.244 | 0.000 | 673647.600 |
| Mg | Weighted median | 5 | -0.801 | 3.200 | 0.802 | -7.073 | 5.470 | 0.449 | 0.001 | 237.503 |
| Mg | Weighted mode | 5 | -1.392 | 3.944 | 0.742 | -9.121 | 6.338 | 0.249 | 0.000 | 565.860 |
| Mg | Simple mode | 5 | 3.100 | 5.156 | 0.580 | -7.006 | 13.205 | 22.187 | 0.001 | 542848.500 |
| se | Inverse variance weighted | 9 | -0.024 | 0.074 | 0.740 | -0.169 | 0.120 | 0.976 | 0.845 | 1.127 |
| se | MR Egger | 9 | 0.128 | 0.238 | 0.607 | -0.339 | 0.595 | 1.137 | 0.713 | 1.812 |
| se | Weighted median | 9 | -0.070 | 0.101 | 0.489 | -0.268 | 0.128 | 0.932 | 0.765 | 1.137 |
| se | Weighted mode | 9 | -0.099 | 0.143 | 0.509 | -0.378 | 0.181 | 0.906 | 0.685 | 1.198 |
| se | Simple mode | 9 | -0.153 | 0.149 | 0.335 | -0.446 | 0.140 | 0.858 | 0.640 | 1.150 |
| Folate | Inverse variance weighted | 2 | 0.167 | 0.378 | 0.657 | -0.572 | 0.907 | 1.182 | 0.564 | 2.478 |
| Fe | Inverse variance weighted | 3 | -0.055 | 0.267 | 0.837 | -0.578 | 0.468 | 0.947 | 0.561 | 1.597 |
| Fe | MR Egger | 3 | 0.138 | 0.747 | 0.884 | -1.327 | 1.602 | 1.148 | 0.265 | 4.965 |
| Fe | Weighted median | 3 | -0.021 | 0.201 | 0.918 | -0.415 | 0.374 | 0.980 | 0.660 | 1.453 |
| Fe | Weighted mode | 3 | -0.356 | 0.215 | 0.239 | -0.776 | 0.065 | 0.701 | 0.460 | 1.067 |
| Fe | Simple mode | 3 | 0.257 | 0.432 | 0.612 | -0.590 | 1.103 | 1.293 | 0.555 | 3.014 |
| P | Inverse variance weighted | 4 | 1.398 | 0.553 | 0.011 | 0.315 | 2.481 | 4.049 | 1.371 | 11.956 |
| P | MR Egger | 4 | 1.309 | 2.682 | 0.674 | -3.948 | 6.566 | 3.701 | 0.019 | 710.250 |
| P | Weighted median | 4 | 1.237 | 0.665 | 0.063 | -0.068 | 2.541 | 3.444 | 0.935 | 12.690 |
| P | Weighted mode | 4 | 1.196 | 0.740 | 0.205 | -0.255 | 2.646 | 3.306 | 0.775 | 14.101 |
| P | Simple mode | 4 | 1.281 | 0.779 | 0.199 | -0.246 | 2.809 | 3.602 | 0.782 | 16.593 |
| β-carotene | Inverse variance weighted | 4 | 0.198 | 0.145 | 0.172 | -0.086 | 0.481 | 1.219 | 0.918 | 1.618 |
| β-carotene | MR Egger | 4 | 0.339 | 1.092 | 0.786 | -1.801 | 2.478 | 1.403 | 0.165 | 11.922 |
| β-carotene | Weighted median | 4 | 0.212 | 0.163 | 0.193 | -0.108 | 0.532 | 1.237 | 0.898 | 1.703 |
| β-carotene | Weighted mode | 4 | 0.117 | 0.218 | 0.629 | -0.310 | 0.543 | 1.124 | 0.734 | 1.721 |
| β-carotene | Simple mode | 4 | 0.113 | 0.229 | 0.657 | -0.336 | 0.562 | 1.119 | 0.714 | 1.754 |
| cu | Inverse variance weighted | 2 | -0.072 | 0.130 | 0.578 | -0.327 | 0.182 | 0.930 | 0.721 | 1.200 |
| zn | Inverse variance weighted | 2 | 0.021 | 0.133 | 0.872 | -0.238 | 0.281 | 1.022 | 0.788 | 1.325 |
| ca | Inverse variance weighted | 7 | -0.190 | 0.761 | 0.803 | -1.682 | 1.302 | 0.827 | 0.186 | 3.676 |
| ca | MR Egger | 7 | 1.347 | 1.303 | 0.349 | -1.208 | 3.902 | 3.846 | 0.299 | 49.487 |
| ca | Weighted median | 7 | 0.754 | 0.720 | 0.295 | -0.657 | 2.165 | 2.126 | 0.519 | 8.719 |
| ca | Weighted mode | 7 | 0.733 | 0.778 | 0.382 | -0.791 | 2.258 | 2.082 | 0.453 | 9.561 |
| ca | Simple mode | 7 | 0.246 | 1.435 | 0.869 | -2.567 | 3.060 | 1.280 | 0.077 | 21.318 |
